# Potential role of contrast-enhanced ultrasound for the differentiation of malignant and benign gallbladder lesions in East Asia: A meta-analysis and systematic review

**DOI:** 10.1097/MD.0000000000011808

**Published:** 2018-08-17

**Authors:** Yuan Cheng, Manni Wang, Buyun Ma, Xuelei Ma

**Affiliations:** a State Key Laboratory of Biotherapy and Cancer Center, West China Hospital, Sichuan University and Collaborative Innovation Center; b Department of Ultrasound, West China Hospital, Sichuan University, Chengdu, People's Republic of China.

**Keywords:** contrast-enhanced ultrasound, diagnosis, gallbladder carcinoma, meta-analysis

## Abstract

**Background:**

The purpose of this study was to systematically review and evaluate the diagnostic accuracy of contrast-enhanced ultrasound (CEUS) in differentiating malignant and benign gallbladder lesions.

**Methods:**

We conducted a comprehensive search on PubMed, Embase, and Cochrane Library for all potential relevant articles published before December 2017. The pooled sensitivity, specificity, diagnostic odds ratio (DOR) and area under the curve (AUC) of summary receiver operating characteristic (SROC) were calculated by Meta-Disc Version 1.4 and STATA 12.0.

**Results:**

Twelve eligible studies were included in our study. A total of 1044 patients were assessed. The pooled sensitivity and specificity were 0.81 (95% confidence interval [CI], 0.77–0.84; inconsistency index [*I*^2^] = 84.5%) and 0.87 (95% CI, 0.85–0.89; *I*^2^ = 94.4%), respectively. The pooled DOR was 58.84 (95% CI, 32.39–106.88; *I*^2^ = 51.9%). The AUC was 0.9371. According to Deek funnel plot asymmetry test, there was no significant publication bias (*P* = .31).

**Conclusions:**

The results yielded from the available evidence suggest that CEUS is a promising and adjuvant imaging technique to conventional ultrasound for the differential diagnosis of benign and malignant gallbladder lesions.

## 1 Introduction

According to the Global Cancer Statistics, gallbladder carcinoma is a relatively rare malignancy worldwide,^[1]^ but it is the most common malignant cancer in the biliary tract, accounting for about 80% to 90%, and the prognosis is poor, with a low 5-year survival rate.^[2]^ The occurrence of gallbladder cancer differs among different geographic distributions and shows high frequency in Asia.^[3]^ Since the symptoms and signs are non-specific, this disease is often diagnosed at a late stage or discovered at the time of cholecystectomy for biliary colic or cholelithiasis. Meanwhile, benign and malignant gallbladder diseases share many same symptoms and signs.^[4]^ Patients who are detected with cancer by accident usually have better prognosis than those who are diagnosed by clinical symptoms.^[5]^ In most cases, patients do not meet the operation criteria, so a 3 to 6 month follow-up is recommended.^[6]^ However, canceration may occur during the entire follow-up. Although several characteristics, like the size of the tumors and the age of patients, are considered helpful to distinguish benign from malignant gallbladder lesions,^[7]^ the accuracy is barely satisfactory without appropriate imaging techniques. During clinical diagnostic process, as an imaging modality, ultrasound is usually firstly used to detect biliary tract diseases. But when it comes to differentiating malignant and benign lesions, the sensitivity remains poor (44%).^[8]^ Therefore, an effective imaging technique for differentiating suspicious malignant gallbladder lesions is quite necessary.

Contrast-enhanced ultrasound (CEUS) is an imaging technique which uses contrast agents (signal enhancer) to inject into the blood circulation. The injected microbubble contrast agents can enhance the contrast between the blood and surrounding tissue.^[9]^ Thus CEUS makes it possible to detect tumor blood flow clearly. Nowadays, CEUS is applied in the examination of several organs like breast, thyroid, kidney, liver, ovary, and especially in the identification of benign and malignant lesions.^[10–14]^ Meanwhile, as the ultrasound contrast agents (UCAs) develop, CEUS has overcome the limitations of conventional ultrasound to a great extent.^[15]^ However, the application of CEUS in identifying gallbladder carcinoma is still under debate. Clinical characteristics are still considered as significant differential indicators for benign and malignant gallbladder lesions. Moreover, in the guideline of European Federation of Societies for Ultrasound in Medicine and Biology (EFSUMB), CEUS is not recommended to help distinguish malignant from benign gallbladder polyps.^[16]^ But in the version of 2017, the EFSUMB guidelines has added a recommendation, according to which CEUS may differentiate chronic cholecystitis from gallbladder carcinoma.^[17]^

Over the past few years, there are several clinical studies to assess the efficacy and accuracy of CEUS in the diagnosis of gallbladder carcinoma, we conducted this study to summarize the available evidence and assess the performance of CEUS in the differentiation between benign and malignant gallbladder lesions.

## 2 Material and methods

### 2.1 Literature search strategy

This study was approved by institutional ethics committee of West China Hospital. Relevant studies published before December 2017 were identified through a comprehensive search of PubMed, Embase, and Cochrane library. The search terms were combinations of the relevant medical subject heading (MeSH) terms, key words and word variants for “gallbladder,” “neoplasm,” and “contrast-enhanced ultrasound.” Title and abstract of each study were reviewed firstly, then full text was read to further screen the articles. In addition, the references of each retrieved article were manually screened to identify other potential eligible studies. The following criteria decided whether these papers were eligible. And disagreements were resolved by a third reviewer. If there were any necessary for further information, we contacted the authors for detail.

### 2.2 Inclusion and exclusion criteria

Inclusion and exclusion criteria were set before the literature search.

Studies were selected if satisfied these criteria:

Clinical studies focused on the diagnostic value of CEUS for the distinction of benign and malignant gallbladder diseases; the gold reference standard for diagnosis was histopathological findings; data were sufficient enough to construct a 2 × 2 contingency table for true positives (TP), false positives (FP), true negatives (TN), and false negatives (FN); informed consents were obtained from each patient and approved by ethics committee; articles written in English or Chinese.

Studies were excluded if met these criteria:

Letters, reviews, editorial articles, or case reports; studies lacked of necessary data.

When the data of 2 articles were from the same medical center with similar patient groups, the article with a larger sample size was selected.

### 2.3 Data extraction

Data extraction was conducted by 2 researchers independently, including the first author's name, publication year of the study, country, mean age of patients, number of patients and lesions, gold reference standard, probe frequency, mechanical index (MI), contrast agents, and contrast modes. In each selected study, true positive (TP), true negative (TN), false positive (FP), and false negative (FN) were collected directly or calculated according to the sensitivity, specificity, positive predictive value (PPV), and negative predictive value (NPV). Divergences were assessed by a third reviewer.

### 2.4 Statistical analysis

All the statistical analyses were performed by STATA 12.0 (Stata Corporation, College Station, Texas) and Meta-Disc Version 1.4 (Unit of Clinical Biostatistics team of the Ramony Cajal Hospital, Madrid, Spain). A summary of sensitivity, specificity, positive likelihood ratios (PLR), negative likelihood ratios (NLR), and diagnostic odds ratio (DOR) were calculated from the TP, FP, FN, and TN of each study, which indicated the accuracy of CEUS in the differentiation of benign and malignant gallbladder lesions. Meanwhile, the summary receive-operating characteristics (SROC) curve was constructed as described by Moses et al^[18]^ to summarize the TP and FP rates. The inconsistency index (*I*^2^) was used to detect the heterogeneity among different studies. *I*^2^ > 50% indicated significant heterogeneity,^[19]^ then we would use a random effect model to continue our analysis.^[20]^ Publication bias was assessed by Deek funnel plot asymmetry test, and *P* > .05 was considered no significant publication bias.^[21]^

### 2.5 Quality assessment

To assess the methodological quality of included studies, Quality Assessment of Diagnostic Accuracy Studies (QUADAS) tool was used by 2 researchers independently, the form of which was constituted of 14 questions.^[22]^ For each item, the study was rated as “yes” (high quality) if reported; “no” (low quality) if not reported; “unclear” if no adequate information was provided. Disagreements were also resolved by a third researcher.

## 3 Results

### 3.1 Study selection

**Figure 1:**
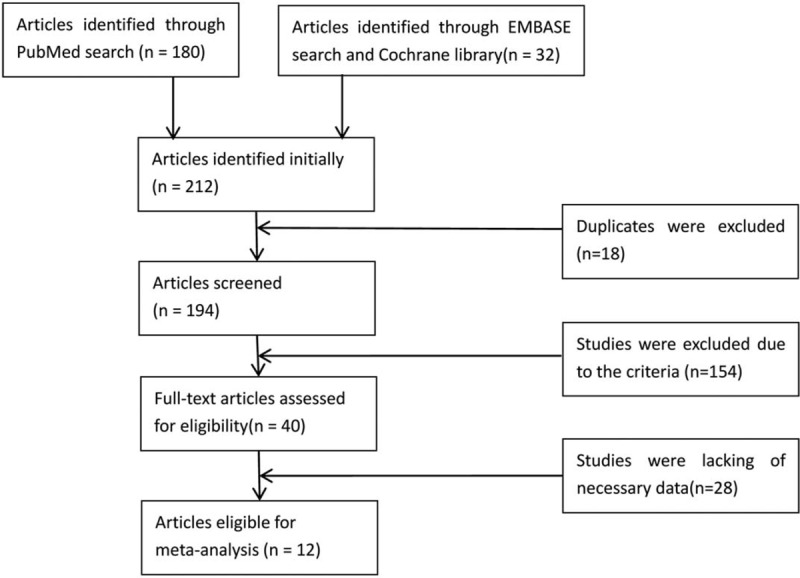
Flowchart of full screening and selection process.

The search process for the meta-analysis is presented in Fig. 1. A total number of 194 articles were included after duplicates were removed. Then we excluded 154 articles according to exclusion and inclusion criteria by scanning the title, abstract, and keywords. After full-text reading, 28 articles were excluded due to missing data. Thus, we finally adopted 12 eligible articles for our meta-analysis.^[23–34]^

### 3.2 Assessment of study quality and publication bias

The methodological quality for each study was assessed by QUADAS. Table 1 presents the results of the evaluation of all those included studies. The item 4 refers that CEUS and standard tests were performed within 3 months. Overall, the quality of the studies was satisfactory.

**Table 1:**
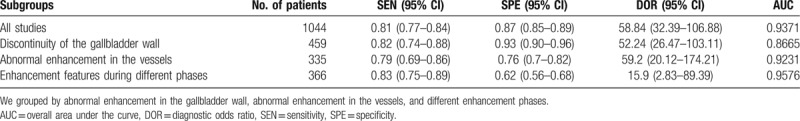
The results of subgroup analysis.

**Figure 2:**
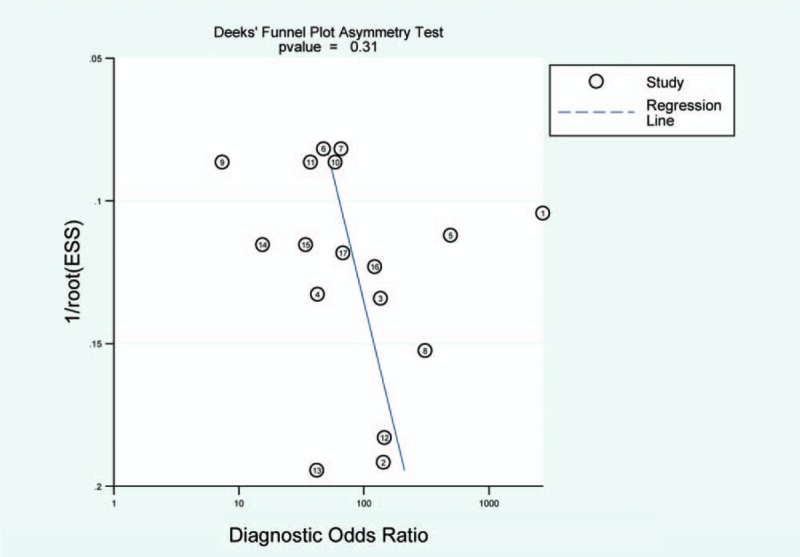
Deek funnel plot asymmetry test.

In our study, the Deek funnel plot asymmetry test was performed to evaluate the publication bias among those eligible studies and the results were shown in Fig. 2. There was no significant publication bias existing in this meta-analysis (*P* = .31).

### 3.3 Study characteristics

The characteristics of the 12 included studies were summarized in Table 2. All of them were published between 2007 and 2017 and written in English. A total of 1044 patients were assessed. The micro-bubble contrast agent used was second generation, SonoVue. In 4 studies, 2 or 3 different enhancement patterns mentioned in the articles showed different sensitivity or specificity. To avoid bias, we included data of these different patterns.

**Table 2:**
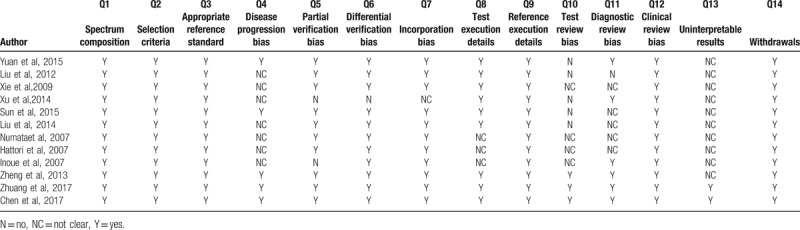
Quality Assessment of Diagnostic Accuracy Studies (QUADAS) questionnaire for the quality assessment of included studies.

### 3.4 Differentiation of benign and malignant gallbladder lesions

**Figure 3:**
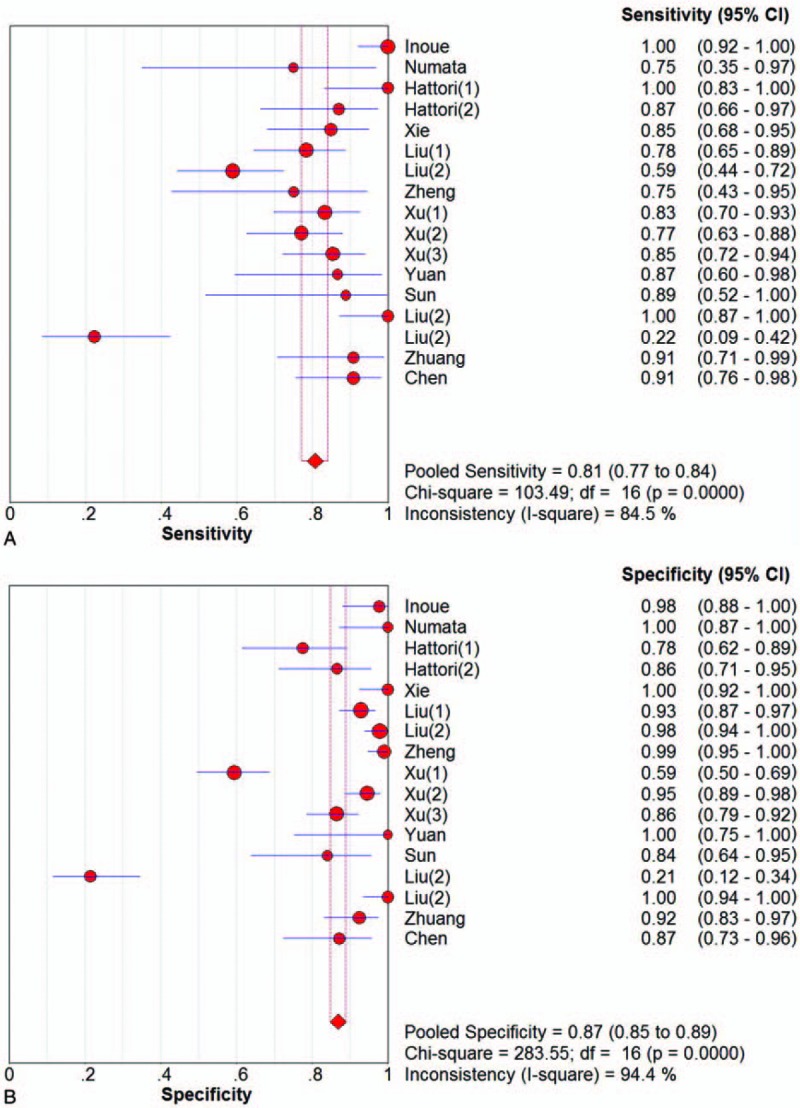
The pooled sensitivity (A) and specificity (B) for the differential diagnosis of benign and malignant gallbladder lesions.

**Figure 4:**
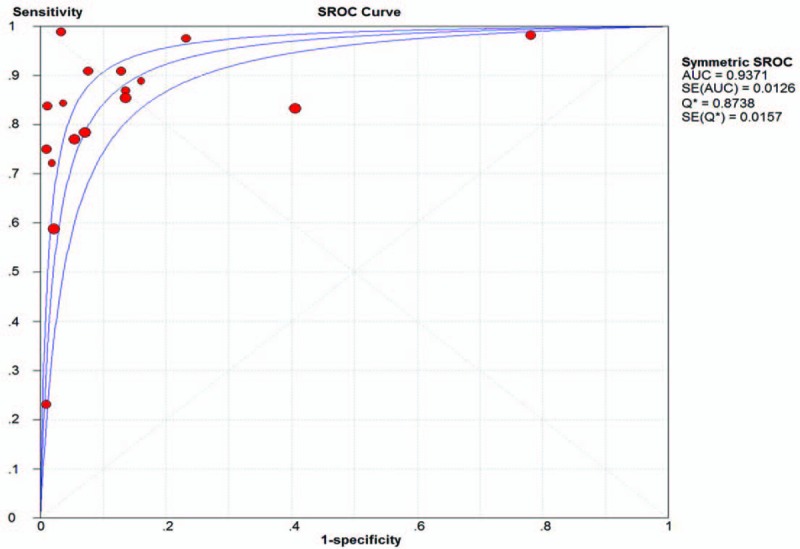
Symmetric summary receiver operating characteristic (SROC) curves.

We used the sensitivity, specificity, PLR, NLR, DOR, and area under the curve (AUC) to evaluate the role CEUS played in the differential diagnosis of benign and malignant gallbladder lesions. The pooled sensitivity and specificity were 0.81 (95% CI, 0.77–0.84; *I*^2^ = 84.5%) and 0.87 (95% CI, 0.85–0.89; *I*^2^ = 94.4%), respectively (Fig. 3A, B). Due to significant heterogeneity, random-effect model was used. The pooled PLR and NLR were 10.43 (95% CI, 4.57–23.83; *I*^2^ = 96.8%) and 0.19 (95% CI, 0.11–0.33; *I*^2^ = 89.6%), respectively. The pooled DOR was 58.84 (95% CI, 32.39–106.88; *I*^2^ = 51.9%). Figure 4 shows the AUC of the summary receiver operating characteristic curve for the value of CEUS in the diagnosis of gallbladder lesions was 0.9371.

### 3.5 Subgroup analysis

As we know, the sensitivity and specificity of CEUS differs due to many factors. Different operators would focus on different features during the whole procedure which might lead to different sensitivity and specificity. Meanwhile, in order to minimize the heterogeneity in our study, we conducted subgroup analyses. All the subgroup analyses are shown in Table 3.

**Table 3:**
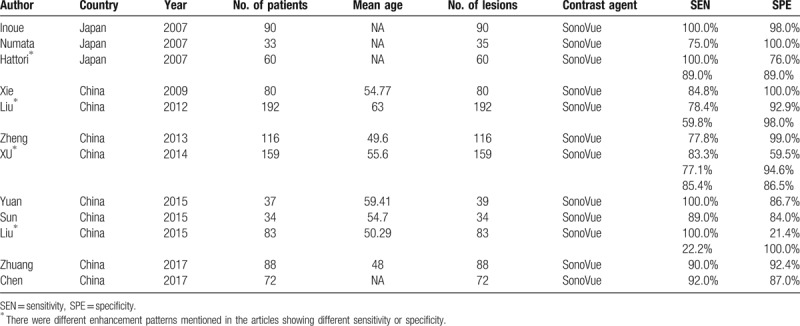
The characteristics of including studies.

#### 3.5.1 Discontinuity of the gallbladder wall

There were 4 studies (Sun et al,^[33]^ Xie et al,^[29]^ Xu et al,^[31]^ Yuan et al^[24]^) mentioned the integrity of the gallbladder wall during enhancement. Other features like the thickness of gallbladder wall were also mentioned in these papers. The pooled sensitivity, specificity and DOR (random-effect model) were 0.82 (95% CI, 0.74–0.88; *I*^2^ = 0.0%), 0.93 (95% CI, 0.90–0.96; *I*^2^ = 69.9%), and 52.24 (95% CI, 26.47–103.11; *I*^2^ = 6.4%), respectively. This sign showed the highest sensitivity and specificity.

#### 3.5.2 Abnormal enhancement in the vessels

When we grouped characteristics by the abnormal enhancement in the vessels, the pooled sensitivity, specificity, and DOR (random-effect model) for identifying malignant gallbladder lesions were 0.79 (95% CI, 0.69–0.86; *I*^2^ = 93.9%), 0.76 (95% CI, 0.70–0.82; *I*^2^ = 98.5%), and 59.2 (95% CI, 20.12–174.21; *I*^2^ = 0.0%), respectively. Gallbladder carcinoma usually showed tortuous and irregular vascular distribution whereas the vessels of gallbladder adenoma might appear in a more regular way like a blossom or a tree.^[24]^

#### 3.5.3 Enhancement features during different phases

The pooled sensitivity, specificity, and DOR (random-effect model) of this subgroup were 0.83 (95% CI, 0.75–0.89; *I*^2^ = 34.1%), 0.62 (95% CI, 0.56–0.68; *I*^2^ = 90.3%), and 15.9 (95% CI, 2.83–89.39; *I*^2^ = 84.5%), respectively. At the peak of enhancement, gallbladder carcinoma might show in hyper-enhancement or iso-enhancement with a relatively low specificity. But “Fast-in and fast-out” could appear during the enhancement^[24,29]^ which might be caused by the abundant blood supply in the malignant lesions.

## 4 Discussion

This meta-analysis which included 12 articles aimed to systematically assess the differential diagnostic performance of contrast-enhanced ultrasound (CEUS) for the gallbladder carcinoma. They all used a second generation contrast agent, SonoVue, and low mechanical index. For the malignant gallbladder lesions, several characteristics could be noticed on the screen, including the destruction of gallbladder wall intactness, infiltration to the surrounding tissue, and branched or tortuous intralesional vessels. However, there are still conflicts in the accuracy of CEUS for identifying gallbladder carcinoma.^[16]^

For now, conventional ultrasound is still a preferred method for the diagnosis of gallbladder diseases, because it's not only less expensive than computed tomography (CT) or magnetic resonance imaging (MRI), without radiation,^[35]^ but also well accessible, with high sensitivity and specificity.^[36]^ However, the difficulty of identifying the perfusion defect and the infiltration to adjacent tissue makes the sensitivity and specificity of conventional ultrasound much lower.^[25]^ Studies found that 61% patients who were suspicious of having mass lesions identified on ultrasound actually had no neoplastic or other mass after the cholecystectomy.^[37]^ CEUS has shown advantages in distinguishing gallbladder carcinoma from motionless sludge, cholesterol polyp, and adenoma^[38,39]^ and already been widely used in abdomen organs especially in the liver.^[15]^ However, experts thought clinical signs and features, such as the diameter of lesions, were more important in the differentiation of benign and malignant gallbladder lesions,^[16]^ which indeed inhibited more investigations into this field. For example, lesion size seemed to be an independent predictor. Diameter of gallbladder lesion ≥2.0 cm showed 100% sensitivity of detecting malignant gallbladder disease with a relatively low specificity.^[29]^ When it was greater than 3 cm, the diagnostic accuracy of malignant lesions still remained 66.7%.^[25]^ However, this was not enough for a malignant diagnosis. Application of CEUS could detect more malignant features of a lesion, such as invasion of surrounding tissues, abnormal vessels, and blood supply. Thus, in order to gather the available evidence, we conducted this meta-analysis.

In our study, the pooled sensitivity and specificity of CEUS for the differential diagnosis between benign and malignant gallbladder lesions were 0.81 and 0.87, respectively, which was much higher than conventional ultrasound (sensitivity, 0.44).^[8]^ And the diagnostic accuracy quantified by AUC of SROC was 0.9371. According to these data, CEUS could be considered as a promising imaging technique in distinguishing benign gallbladder lesions from malignant ones. And no similar study has been conducted during our search.

We noticed that there was an obvious difference of sensitivity and specificity among different imaging performances. According to our subgroup analysis, the discontinuity of gallbladder wall showed the highest sensitivity and specificity (0.82 and 0.93, respectively). Discontinuity and infiltration to the surrounding tissue of the gallbladder wall were difficult to be detected through conventional ultrasound, which were usually the clues for malignant lesions. The study of Xie et al^[29]^ demonstrated that the destruction of the gallbladder wall intactness on CEUS was the best indicator of malignant lesions with the highest sensitivity and specificity (0.85 and 1.00, respectively). Malignant and benign gallbladder wall showed different thickness which were 17.3 ± 5.2 (6–30) mm and 8.6 ± 5.1 (4–26) mm, respectively.^[31]^ However, when thickened gallbladder wall was viewed on conventional ultrasound, it was always difficult to distinguish gallbladder carcinoma from chronic cholecystitis.^[40]^ However, inner layer discontinuity, which was much more pronounced in CEUS, was helpful to identify malignant thickened gallbladder wall. Thus, the destruction of gallbladder wall intactness on CEUS suggested the high possibility of malignant gallbladder lesions and thickened gallbladder wall might increase this possibility.

Abundant intralesional blood flow usually suggests the possibility of malignancy. Although traditional ultrasound has shown progress in detection of large blood vessels,^[41,42]^ it stills gets limitations on tracking small vessels, especially those inside masses. CEUS showed a much higher blood flow detection rate than conventional ultrasound (0.92 vs 0.42, *P* < .001).^[32]^ When we grouped by abnormal blood flow in lesions, our study showed the pooled sensitivity and specificity were 0.79 and 0.76, respectively. Gallbladder carcinoma usually showed tortuous vascular distribution, whereas the vessels of gallbladder adenoma distributed like a blossom or a tree^[24]^ which were more regular. Branched or linear intralesional vessels on CEUS might also suggest the possibility of malignancy. Combined with our clinical experience and research conducted by Numata et al,^[30]^ irregular vascular distribution of the lesion was relatively more common in gallbladder carcinoma. The majority of malignant lesions are hypoenhancing while most solid benign lesions are homogeneous enhancing or hyperenhancing during enhancement phases.^[43]^ These 4 studies in this subgroup all shared high sensitivity and low specificity. When compared malignant gallbladder lesions with benign ones, the contrast arrival time and the time to peak enhancement were significantly shorter in the latter (sensitivity, 0.89; specificity, 0.63).^[33]^ The time to peak enhancement (>20 seconds) and wash-out time (time to hypo-enhancement, <35 seconds) might suggest high possibility of malignancy in the differential diagnosis though its sensitivity and specificity were relatively low.^[25,33]^ However, either malignant and benign gallbladder lesions was mostly hyperenhanced or hypo-enhanced in the arterial phase,^[29]^ which meant it could not be an promising indicator for identifying gallbladder malignancy. Thus, according to our subgroup analysis, whether blood flow in the gallbladder lesions or enhancement features during different phases could be helpful in the differential diagnosis of gallbladder cancer needed to be further investigated.

During our research, we found that contrast-enhanced harmonic endoscopic ultrasonography (CEH-EUS) is a useful method in the diagnosis of digestive system diseases, but studies in this area especially in gallbladder diseases are limited.^[44]^ Three articles demonstrated the diagnostic value of CEH-EUS in the differentiation of benign and malignant gallbladder lesions.^[30,45,46]^ There were 159 patients and the mean age was 60.2. The pooled sensitivity and specificity were 0.88 (95% CI, 0.77–0.95, *I*^2^ = 22.9%) and 0.96 (95% CI, 0.90–0.99, *I*^2^ = 55.5%), respectively. The heterogeneity was not significant. The pooled PLR, NLR, DOR, and AUC were 16.55, 0.16, 188.66, and 0.9787, respectively. These data indicated that CEH-EUS was quite promising. Compared with conventional ultrasound, the images of CEH-EUS could demonstrate the extent and depth of carcinoma invasion much better. However, whether CEH-EUS is better than CEUS needs to be further discussed.

We found significant heterogeneity in our study. In order to minimize the heterogeneity among those included studies, we conducted subgroup analyses. Unfortunately, the heterogeneity still existed. Thus, a random-effect model was used. We thought different operators, machines, and observer variability might lead to this heterogeneity. Meanwhile, according to results of the Deek funnel plot, no publication bias was detected.

There are still several limitations in our study. Firstly, the number of included studies and patients was limited. We assumed that according to the guideline (version 2011) from EFSUMB, CEUS was not recommended in differentiating malignant and benign gallbladder polyps, which might limit clinical studies to investigate the usefulness of CEUS in identifying malignant gallbladder lesions. Secondly, the included 12 studies all came from Asian area. The incidence rate of gallbladder carcinoma in Latin America and Asia is significant high, while it is low in the United States and most Western and European countries.^[47]^ Thus, various geographic patterns for gallbladder cancer might cause some bias in the final analysis.

In conclusion, our results suggest that CEUS is a promising and adjuvant imaging technique to conventional ultrasound for the differential diagnosis between malignant and benign gallbladder lesions. Discontinuity of gallbladder wall and infiltration to the surrounding tissue during enhancement suggest high possibility of malignancy. Tortuous intralesional vessels and thickened gallbladder wall may increase that possibility. Whether different enhancement features, like the time to peak or wash-out time within 35 seconds, still remain unsure for the differential diagnosis. Thus, more researches are needed to be done to provide more valid evidence, especially some quantitative data. This research can help not only assure the role CEUS plays in the differential diagnosis between malignant and benign gallbladder lesions but also update the guidelines.

## Author contributions

**Conceptualization:** Buyun Ma, Xuelei Ma.

**Data curation:** Yuan Cheng.

**Formal analysis:** Yuan Cheng.

**Methodology:** Yuan Cheng.

**Software:** Yuan Cheng.

**Supervision:** Xuelei Ma.

**Validation:** Buyun Ma, Xuelei Ma.

**Writing – original draft:** Yuan Cheng.

**Writing – review & editing:** Manni Wang.
